# The Fate of Bacteriophages in Recirculating Aquaculture Systems (RAS)—Towards Developing Phage Therapy for RAS

**DOI:** 10.3390/antibiotics8040192

**Published:** 2019-10-24

**Authors:** Gabriel M.F. Almeida, Kati Mäkelä, Elina Laanto, Jani Pulkkinen, Jouni Vielma, Lotta-Riina Sundberg

**Affiliations:** 1 Department of Biological and Environmental Science, Nanoscience Center, University of Jyväskylä, 40014 Jyväskylä, Finlandkati.j.makela@jyu.fi (K.M.); elina.laanto@jyu.fi (E.L.); 2 Faculty of Biological and Environmental Sciences, Molecular and Integrative Biosciences Research Programme, University of Helsinki, 00014 Helsinki, Finland; 3 Natural Resources Institute Finland, Production Systems, 40500 Jyväskylä, Finland; jani.t.pulkkinen@luke.fi (J.P.); Jouni.Vielma@luke.fi (J.V.)

**Keywords:** aquaculture, bacteriophage, biofilter, disease, phage therapy, RAS, recirculating aquaculture systems

## Abstract

Aquaculture production has increased tremendously during the last decades, and new techniques have been developed, e.g., recirculating aquaculture systems (RAS). In RAS, the majority of water volume is circulated via mechanical and biological filters and reused in the tanks. However, the prevention and treatment of diseases in these systems are challenging, as the pathogens spread throughout the system, and the addition of chemicals and antibiotics disrupts the microbiome of the biofilters. The increasing antibiotic resistance has made phage therapy a relevant alternative for antibiotics in food production. Indeed, as host-specific and self-replicating agent they might be optimal for targeted pathogen eradication in RAS. We tested the survival and spread of *Flavobacterium columnare* -infecting phage FCL-2 in recirculating aquaculture fish farm with rainbow trout (*Oncorhynchus mykiss*) in a fully controlled study. After a single addition, phage persisted in water samples collected from tank, fixed bed, moving bed, and aeration unit up to 14 days, and in the water of rearing tanks, rainbow trout mucus, and bioreactor carrier media from the fixed and moving bed biofilters for 21 days. Furthermore, phage adsorbed preferentially to moving bed carrier media, which contained biofilm attached and from which higher phage numbers were recovered. This study shows phages as a potent strategy for maintaining biosecurity in RAS systems.

## 1. Introduction

Aquaculture production is an important source of a protein destined for human consumption. It is an expanding industry with many strategies employed to improve efficiency and environmental impact. During recent years, a growing interest in recirculating aquaculture systems (RAS) has appeared [1,2]. Water in RAS is reused after being treated, dramatically reducing the amount of water needed for fish farming in aquaculture sites. Confining large populations of aquatic animals are a risk factor when considering infectious diseases, and aquaculture rearing units have been shown to favor pathogens and virulence increase [3,4,5]. The reuse of water in RAS may pose a biosecurity challenge for infectious diseases, as the removal of pathogens or chemical residues from the tanks is difficult, increasing the unnecessary exposure of aquatic animals to both [6]. Many of the pathogens that threaten aquaculture are bacteria, and the diseases are treated with antibiotics. Antibiotic leakage to the environment can lead to environmental and health issues (i.e., alterations in microbial communities and selection for resistance against antibiotics) [7]. For example, antibiotic resistance genes are enriched in sediments associated with aquaculture sites [8,9]. Thus, means to implement the One Health approach (https://www.who.int/features/qa/one-health/en/) in aquaculture production need to be developed.

One possible alternative to antibiotics and a solution to the antimicrobial resistance crisis and One Health approach is the use of (bacteria) phage therapy. Phages are viruses that specifically infect only bacteria, without causing harm to surround microbiota or eukaryotic cells. Phage therapy is not a novel concept, as it has been used for almost a century to treat bacterial infections in humans [10,11]. It has also been applied to aquaculture related bacterial pathogens, experimentally, and in practice [12,13]. So far, there is no information about phage use in RAS, but this type of system can be considered an ideal environment for phage therapy applications [14]. The water recirculation process is an advantage for phage delivery since, in theory, phage would remain in the system for long time periods for being small enough to pass through filters and other barriers, recirculating freely with the water. This would enable prolonged protection via extending the time animals are exposed to phage, while also favoring phage evolution in response to phage-resistant bacteria, which has been documented in open aquaculture systems [15]. In addition, whereas chemicals and antibiotics might influence the microbiome composition of biological filters [16], host-specific phages infect only their target bacteria. However, phage stability over time on RAS farms, phage distribution in the tanks, and how the different treatment units affect phage survival have not been tested.

In this work, we investigated phage dynamics in RAS research fish farm with rainbow trout (*Oncorhynchus mykiss*). As a model, we used *Flavobacterium columnare* -infecting phage FCL-2, which has been isolated from the same fish farm years earlier and shown to be suitable for phage therapy in our earlier studies [12,17]. Phage solution was added to the water of three rearing tanks, and phage numbers were measured over time from different locations of each system, on the fish mucus, and on plastic carrier media used to collect biofilm in the moving and fixed-bed bioreactors. We demonstrate phage persistence in a RAS for three weeks after a single phage exposure. Phage concentration was highest in the plastic carrier media, indicating that bacterial biofilm might be a substrate where phage preferentially enrich. Additionally, phage presence did not alter water quality parameters nor resulted in fish mortality. The use of phage therapy on RAS can be effective since only one phage dose is sufficient for persistence and spread over the whole system without being destroyed by the water treatment processes.

## 2. Results

### 2.1. Phages Persist in RAS for Up to Three Weeks

**Figure 1:**
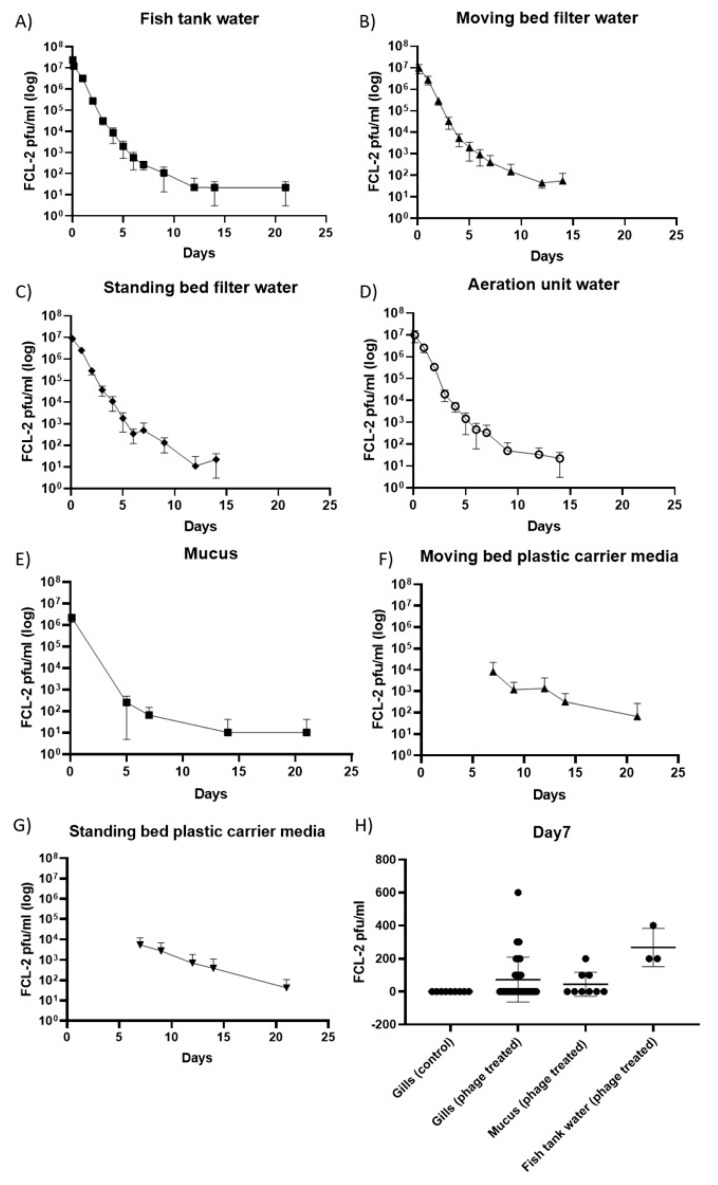
Phage persistence over time in the recirculating aquaculture system (RAS) tanks and fish. Phage FCL-2 titers (pfu/mL) on (**A**) the fish tank water, (**B**) the moving bed water, (**C**) the fixed-bed water, (**D**) the aeration unit water, (**E**) fish mucus, (**F**) the moving bed plastic carrier media, (**G**) the fixed-bed plastic carrier media, (**H**) on fish gills (seven days after phage exposure). In **A**, **B**, **C,** and **D,** each tank was sampled once until day seven and in triplicates from day nine onwards. In **E,** three fish per tank were sampled in the first and fifth day, then three fish per control tank, and ten fish per phage-treated tank were measured from day seven onwards. In **F** and **G,** five carrier media pieces per tank were sampled in all time points. In **H,** three fish from each control tank and ten fish from each phage treated tank were measured.

Phages were recovered from all sampled components of the RAS system up to 14 days, and from the water from rearing tanks, fish mucus and carrier media from the fixed and moving bed biofilters for 21 days after a single phage exposure (Figure 1A–G). In all samples, a similar trend was observed with the phage numbers decaying faster in the first days then becoming more stable at the end. Phage titers were higher in filter carrier media samples, suggesting that bacterial biofilm may have a role in phage enrichment. On day seven, the phage presence on fish gills was tested. Phage titers in gills were similar to the ones found in mucus and slightly lower than in water (Figure 1H). No infective phage particles appeared in samples taken from the control tanks over the course of the experiment.

No phages capable of infecting *Flavobacterium columnare* strain B185 were detected from water or mucus samples collected at time zero. Also, no *F. columnare* was isolated from fish gills sampled at time zero. These negative controls, allied to the data from control tanks, support the idea that phages found over time were derived from the original lysate and not from the environment or natural infections during the course of the experiment.

Water parameters were measured over the course of the experiment, and no significant changes were detected between control and phage treated RAS (Table 1). No fish mortalities nor decreased feed intake rate were observed in the RAS units treated with phage.

### 2.2. Attachment of Phages to Filter Pellets Is Dependent on Biofilm

**Figure 2:**
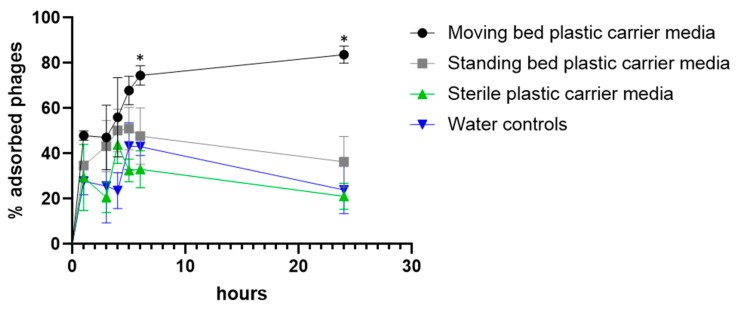
Adsorption of phage to plastic carrier media. Each condition was tested in triplicates. Unpaired t-tests were used for comparing the controls and tested conditions. * Statistical difference found (exact p values are mentioned in the text).

As the data collected from the RAS experiment indicated, phages were preferentially enriched in the plastic carrier media of fixed and moving bed biofilters. Therefore we decided to test phage adsorption in this material in vitro directly. Sterile carrier media and media collected from control (no phage) tanks were exposed to phage, and the number of free phages was determined in the supernatant. It should be noted that the media collected from fish tanks were colonized by biofilms, while autoclaved media were clean and sterile. The percentage of attached phage was calculated from the titer (pfu/mL) of the free phage particles in the water at each time point (which in turn was used to calculate the corresponding number of phage particles attached to the pellets). (Figure 2). Although phage inactivation was not tested, it is unlikely to have affected the results since phage FCL-2 is considerably stable over time. It is clear that phage adsorbed preferentially to moving bed bioreactor media. After six hours, more phages were adsorbed to moving bed than to fixed bed media and sterile media (*p* = 0.011 and *p* = 0.000033, respectively). This persisted up to 24 hours after exposure (*p* = 0.000013 when compared to fixed-bed filters and *p* < 0.000001 when compared to sterile carrier media).

## 3. Discussion

Bacterial disease epidemics need antibiotic treatment also in fish production, but the legislation related to antibiotic use in aquaculture differs between countries. Nevertheless, antibiotic use influences microbial ecology and resistance evolution also outside fish farming [7,18,19], affecting environmental and food safety. At the time of the ‘post-antibiotic era’ declared by the WHO, interest towards sustainable tools that reduce the need for antibiotics is increasing. Phages infecting aquaculture pathogens such as *Flavobacterium* and *Vibrio* species have already been isolated [14,20,21]. 

The use of RAS is advantageous to other aquaculture methods when considering water use and nutrient discharges. However, it is also a vulnerable environment for outbreaks of infectious diseases. RAS has been considered to be an optimal environment for the use of phage therapy against bacterial diseases [14], but so far, phage distribution and stability have not been tested on these systems. Here we show that after a single phage addition, the phage can be re-isolated from the RAS units at a fish farm for up to three weeks. Phages were re-isolated from water collected from different points in the system, from fish mucus and gills, and also from plastic carrier media used in biofilter units. Phage persistence was longest in the rearing tank water, fish mucus, and biofilm media. This indicates that interactions with the tank microbiome or with filtration and water treatment systems are not deleterious for phage presence. Furthermore, and perhaps more importantly, phage addition did not cause alterations in water quality nor in fish health, demonstrating it is a safe treatment in the fish farming system. However, detailed knowledge of the applied phage needs to be available to consider its suitability as a treatment [22]. 

Phage retention in animal mucus has been shown previously and associated with the presence of Ig-like domains in the phage structural proteins [23]. We have already shown that phages FCL-2 and T4 are held for up to a week in rainbow trout mucus in open flow-through systems, and that FCL-2 retention has a protective effect against *Flavobacterium columnare* infections [17]. Since in this study, phage FCL-2 was present in the water at all time points due to water recirculation in the system, phage enrichment in mucus was not evident although it lasted longer than in the flow-through systems [17]. Longer persistence of phages in mucus and water suggest that protection against infections will also be maintained for longer periods, which could reduce or delay the need for antibiotic treatments.

The rearing water quality in RAS systems relies on the capacity of biofilters to remove ammonia, yet a vast portion of bacteria species in the biofilters are other, mainly heterotrophic bacteria [24,25]. Surprisingly, the plastic media colonized by biofilms in the moving and fixed bed biofilters contained more phages than the rest of the RAS system. Indeed, in vitro testing revealed that biofilm was needed for phage retention in the pellets. While we did not explore this adhesion in more detail, it may be caused by unspecific interactions with the extracellular matrix (mainly exopolysaccharides) protecting the biofilm [26], similarly to what was observed to occur between phages and mucins. Yet, the mechanism by which the phages attach to the microbial biofilm remains unclear. Another possibility could be the presence of the *Flavobacterium* host in the biofilter microbiome. Previous studies on the RAS biofilter microbiome have shown the presence of *Flavobacterium spp* in the biofilters [24], but, although this possibility cannot be excluded, we did not detect *F. columnare* during the experiment. However, our in vitro results suggest a specific microbiome plays a role in phage adhesion in biofilters, as adhesion was significantly higher in moving bed carrier media compared to fixed bed media. This may have also been caused by quantitative differences in the biofilm of these two filter materials. 

In conclusion, we have shown that a phage can persist in RAS units for extended periods of time, and the water treatment processes or system components are not a threat to viral particles or vice versa. Components of the system such as biofilter carrier media and fish mucus may have a positive impact in phage persistence, by selectively enriching phages compared to the water, and slowly releasing them over time. This might be enough to delay the onset of a bacterial disease outbreak by keeping the infective bacterial doses low due to phage infections. Furthermore, by selecting phages targeting unwanted contaminants (pathogens or other bacteria), this approach could be used in developing phage-based techniques in managing the RAS biofilter microbiome. For example, geosmin compounds accumulate in fish tissue, causing unwanted earthy taste and odor [27]. Phages infecting geosmin-producing *Streptomyces* species have already been shown to be capable of reducing geosmin production [28] and could be used in RAS systems to control product quality.

## 4. Material and Methods

### 4.1. Experimental Site

**Figure 3:**
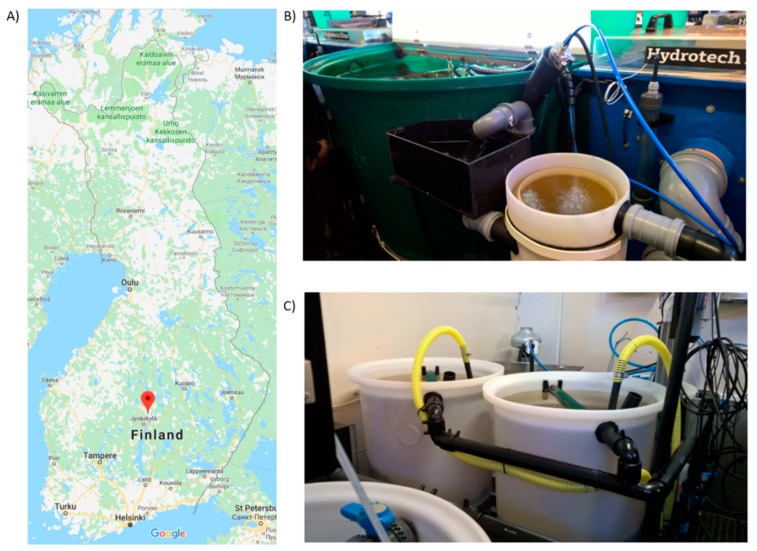
RAS farm location and an example of the fish tanks used. (**A**) Fish farm location in Laukaa, Finland. (**B**) Fish tank (in green) and solids removal units. (**C**) Fixed and moving bed biofilters.

This study was performed at the Laukaa fish farm of the Natural Resources Institute Finland. Six individual RAS units were used, each containing of a 500 L fish tank, a swirl separator, a drum filter, a 150 L fixed and a moving bed biofilter (each filled with 70 L of plastic carrier media, RK Bioelements), and a trickling filter for carbon dioxide removal. In fixed-bed bioreactors, the carrier media lies static in the bottom of the reactor, whereas in moving bed bioreactors, the carrier media is agitated continuously with air. Each RAS unit had a total water volume of 890 liters, with a water flow of 720 liters per hour and water renewal of 150 liters per day (resulting in the turnover rate of the entire water volume in six days). Each tank was stocked with 270–280 rainbow trout (*Oncorhynchus mykiss*) (mean 50 g), and fish were fed 300 g·d^−1^. Fish health and feed intake rate were monitored daily. The location of the fish farm and an example of the RAS system (fish tank, solids removal units, and moving and fixed bed bioreactors) can be seen in Figure 3.

### 4.2. FCL-2 Phage 

FCL-2, a myophage infecting *Flavobacterium columnare*, was used as the model. It has been isolated from the same fish farm where this experiment was performed and shown to be efficient as a phage therapy agent in laboratory conditions [12,17,29]. The phage stock used was prepared by infecting host cultures supplemented with mucin, as described previously [17]. Briefly, the supernatant of overnight *F. columnare* strain B185 cultures made in 0.5× Shieh media supplemented with 0.1% purified porcine mucin (Sigma, USA) were transferred to sterile flasks and infected with FCL-2. Twenty-four hours later, the whole cultures were centrifuged, and the supernatant filtered to obtain a sterile high-titers phage lysate. 

Phages were quantified by the double-agar overlay method. Three hundred microliters of overnight cultures of *F. columnare* strain B185 were added to three milliliters of soft-Shieh agar supplemented with 0.1% mucin, and the mixture was added to the top of Shieh-agar plates. Then, dilutions of the samples were added as drops to the top of the mixture and phage plaques were counted two days later. 

### 4.3. Phage Treatment and Sampling

Before starting the experiment, water and fish mucus samples were collected and tested to guarantee that no pre-existing *F. columnare* or phage capable of infecting *F. columnare* were already present at the systems. Fish gills were also sampled to test for the presence of *F. columnare*. Phage presence was verified by plating the samples on fresh lawns of *F. columnare* strain B185 made in soft-agar-Shieh while *F. columnare* presence was verified by plating the water samples in Shieh-agar containing tobramycin. 

For phage treatment, water flow was stopped in six RAS units. Then 470 milliliters of phage FCL-2 lysate (2 × 10^10^ pfu/mL) was added to the fish tanks (*n* = 3). The final phage concentration in each tank was 1 × 10^7^ pfu/mL at time zero. An equal volume of sterile media was added to control tanks (*n* = 3). After one hour, water samples were collected, and water flow was restarted. Tank water, fish mucus, and plastic carrier media from moving and fixed beds were sampled periodically. Water samples were taken from the fish tank and from the moving and fixed beds and from the trickling filter (aeration unit). Fish were removed from the tanks, euthanatized with an overdose of benzocaine, and skin mucus was scrapped with a glass slide. Sterile water was added to the mucus samples, to a final volume of one milliliter. Plastic media from the moving and fixed beds were collected and kept in falcon tubes immersed in water from the respective bed. All samples were preserved by the addition of chloroform until being processed. 

### 4.4. Ethics

Fish handling was conducted according to the Finnish Act on the Use of Animals for Experimental Purposes, under permission ESAVI/8187/2018 granted for Lotta-Riina Sundberg by the National Animal Experiment Board at the Regional State Administrative Agency for Southern Finland.

### 4.5. Water Quality Monitoring

Water quality was monitored in the fish tanks with an online monitoring system consisting of a spectrometer probe measuring total organic carbon, a pH probe, and an optical oxygen probe. Total ammonia nitrogen (TAN), nitrite, and nitrate were analyzed spectrophotometrically once a week from the tank outlet water. 

### 4.6. Adsorption Tests on Plastic Carrier Media

Clean autoclaved plastic media and media collected from the moving and fixed beds of control (no phage) fish tanks were used for testing phage adsorption and retention in vitro. The media were added to 50 mL falcon tubes and covered with nine ml of tank water. After one hour gently shaking, 1 ml of a phage solution (2 × 10^5^ pfu/mL) was added, and the tubes returned to the shaker. 10 μL samples were collected from the tubes periodically, and added into 990 μL of Shieh medium and a drop of chloroform, and maintained on ice until the phage titers were determined. 

### 4.7. Statistical Analysis 

Data analysis was made using the GraphPad software version 8.0.1 (GraphPad Software, San Diego, USA). Unpaired t-tests were employed for comparing tested conditions in the appropriate datasets.

## 5. Conclusions

Although other phage species must be tested and the effect of phage held in the system on bacterial outbreaks evaluated, our results highlight the relevance of phage-based techniques in developing healthy RAS systems. Phages could be applied as a treatment to ongoing infections in a conventional phage therapy approach, but the potential for preventive phage therapy could be more interesting. By enriching the RAS tanks with the right type of phage, aquatic animal farmers could achieve long-term protection from common aquaculture bacterial diseases, protecting their production while at the same time avoiding the costs and risks of antibiotic use.

## Figures and Tables

**Table 1:** Mean (±SD) water quality parameters of recirculating aquaculture system (RAS) control units and phage-treated units. TAN = total ammonia nitrogen, NO2-N = Nitrite, NO3-N = Nitrate, TOC = total organic carbon.

Parameter	Control Units	Phage-Treated Units
TAN (mg·L^−1^)	1.31 ± 0.11	1.20 ± 0.23
NO2-N (mg·L^−1^)	0.53 ± 0.01	0.57 ± 0.05
NO3-N (mg·L^−1^)	42.4 ± 0.2	43.1 ± 1.2
pH	7.2 ± 0.0	7.2 ± 0.0
T (°C)	15.5 ± 0.3	15.2 ± 0.3
O2 (mg·L^−1^)	7.3 ± 1.0	8.7 ± 0.9
TOC (mg·L^−1^)	14.4 ± 4.6	17.6 ± 4.0
